# Supplementary figures and images for: SAFE-CAST: secure AI-federated enumeration for clustering-based automated surveillance and trust in machine-to-machine communication
Source: PeerJ Comput Sci. 2025 Jan 2;11:e2551. doi: 10.7717/peerj-cs.2551 (PMC11784817; doi:10.7717/peerj-cs.2551)

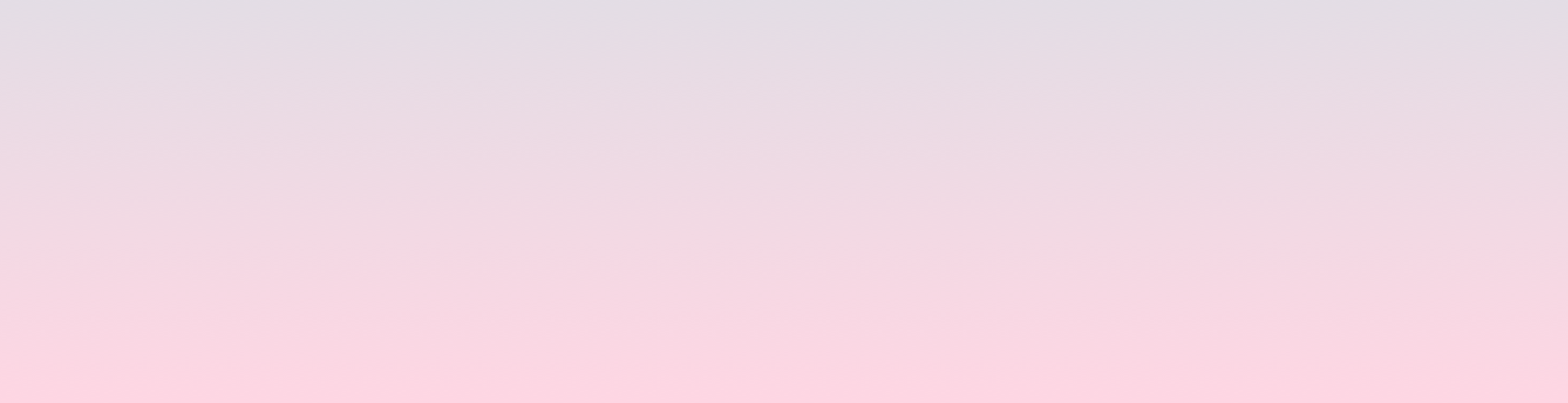

Supplement: Supplemental Information 1 [file peerj-cs-11-2551-s001.zip › code/img1/bg.png]

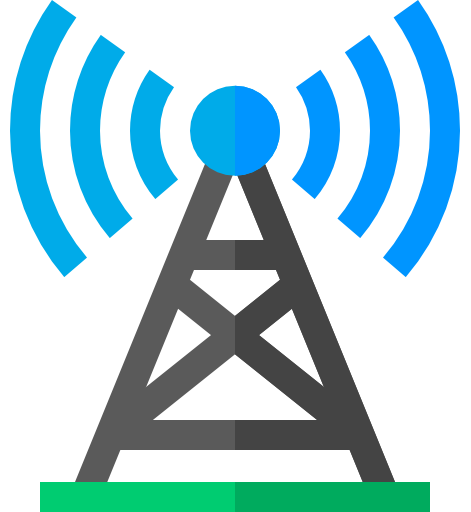

Supplement: Supplemental Information 1 [file peerj-cs-11-2551-s001.zip › code/img1/BS.png]

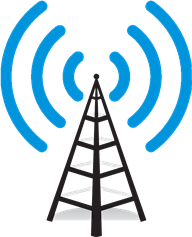

Supplement: Supplemental Information 1 [file peerj-cs-11-2551-s001.zip › code/img1/BS1.png]

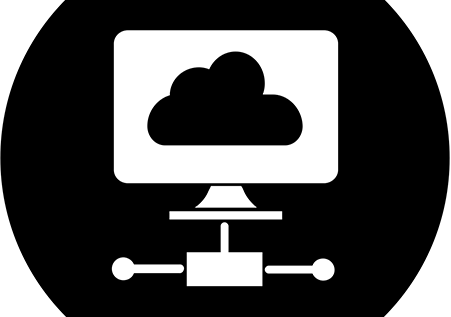

Supplement: Supplemental Information 1 [file peerj-cs-11-2551-s001.zip › code/img1/Cloud.png]

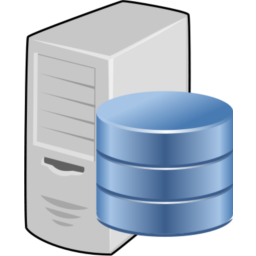

Supplement: Supplemental Information 1 [file peerj-cs-11-2551-s001.zip › code/img1/Edge.png]

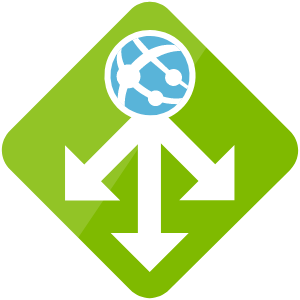

Supplement: Supplemental Information 1 [file peerj-cs-11-2551-s001.zip › code/img1/MA.png]

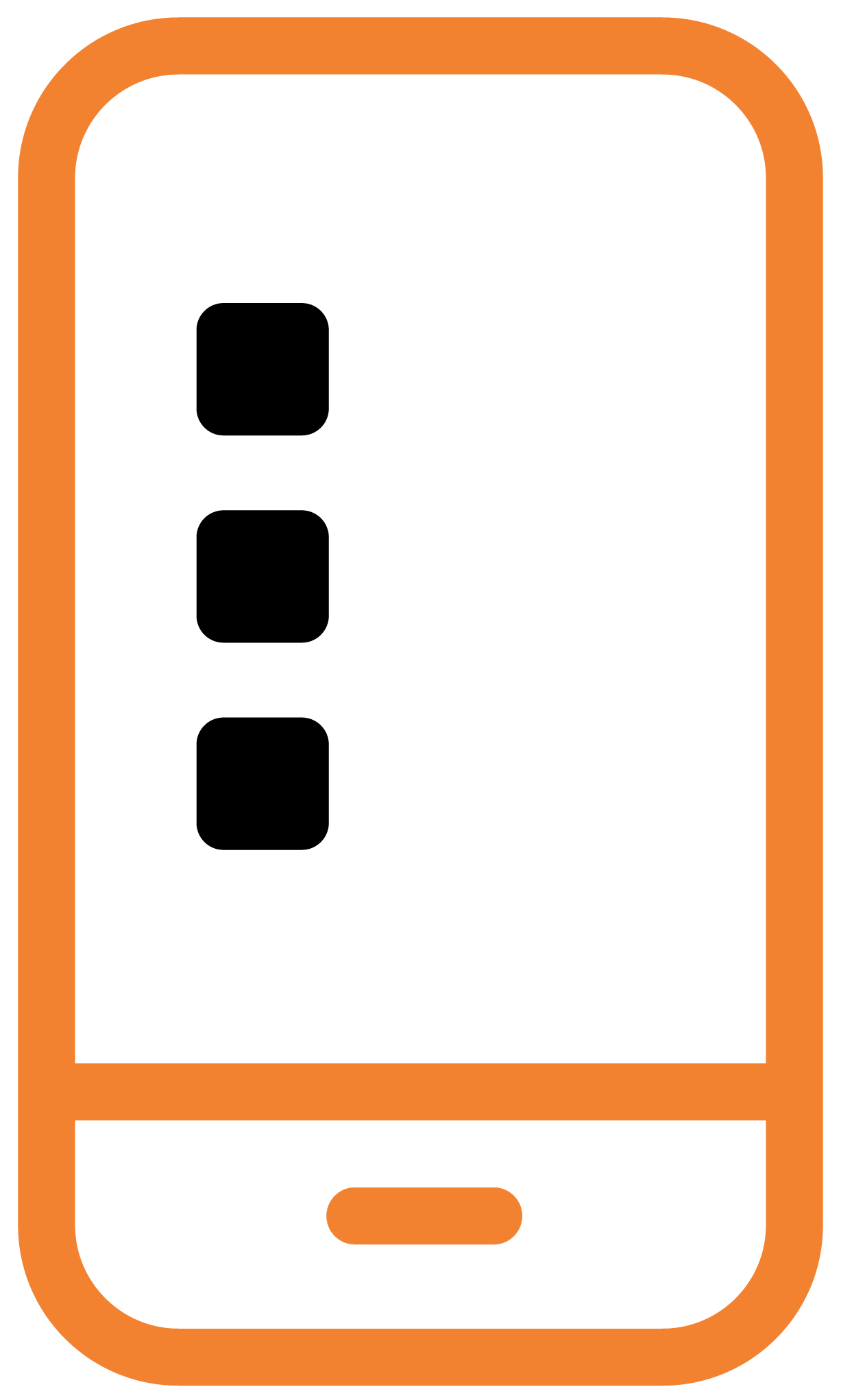

Supplement: Supplemental Information 1 [file peerj-cs-11-2551-s001.zip › code/img1/MACHINE.png]

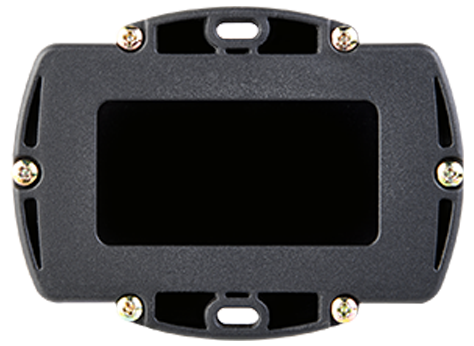

Supplement: Supplemental Information 1 [file peerj-cs-11-2551-s001.zip › code/img1/MACHINE1.png]
